# Myopic axial elongation in school children and the COVID-19 lockdown in Russia: The Ural Children Myopia Study

**DOI:** 10.1371/journal.pone.0279020

**Published:** 2023-01-25

**Authors:** Mukharram M. Bikbov, Gyulli M. Kazakbaeva, Albina A. Fakhretdinova, Azaliia M. Tuliakova, Ellina M. Rakhimova, Songhomitra Panda-Jonas, Leisan I. Gilemzianova, Liana A. Garipova, Dinar A. Khakimov, Jost B. Jonas

**Affiliations:** 1 Ufa Eye Research Institute, Ufa, Russia; 2 Ufa Eye Institute, Ufa, Russia; 3 Department of Ophthalmology, University of Heidelberg, Heidelberg, Germany; 4 Privatpraxis Prof Jonas und Dr Panda-Jonas, Heidelberg, Germany; 5 Department of Ophthalmology, Medical Faculty Mannheim, Heidelberg University, Mannheim, Germany; 6 Institute of Molecular and Clinical Ophthalmology Basel, Basel, Switzerland; Aravind Eye Hospital and Post Graduate Institute of Ophthalmology, INDIA

## Abstract

**Background:**

To explore an influence of the COVID-19-related lockdown on ocular axial elongation in school children in Russia.

**Methods:**

The participants of the school-based Ufa Children Myopia Study in Ufa/Russia underwent, at baseline in 2019/2020 before the COVID-19 outbreak and after a COVID-19-related lockdown, a detailed interview and ophthalmological examination including laser interferometric biometry for axial length measurement.

**Results:**

The study included 461 children (age:10.7±2.1 years;range:6.8–16.9 years). The mean follow-up was 1.41±0.33 years. Mean axial length at baseline was 23.96±0.95mm and 23.94±0.95mm in the right and left eyes, respectively. During the study period, annual axial elongation (right/left eyes) was 0.19±0.17mm/0.19±0.22mm. Before the COVID-19 lockdown, the age-dependent coefficient for axial length (ADCAL) for the right/left eyes was 0.21mm (95%CI:0.17,0.25)/0.20mm (95%CI:0.16,0.24). In children younger than 9.6 years (n = 157), annual axial elongation (right eyes) during the study period was larger than the ADCAL before the COVID-19 outbreak (0.29 mm (95%:0.00,0.66) versus 0.21 mm (95%CI:0.02,0.41)). In the groups aged 9.6 to 11.4 years (n = 148) and aged >11.4 years (n = 156), annual axial elongation during the study period was comparable to the ADCAL before the COVID-19 outbreak (0.18mm (95%CI:-0.07,0.46) versus 0.22mm (95%CI:-0.05,0.48), and (0.09mm (95%CI:-0.15,0.34) versus 0.14mm (95%CI:0.00,0.28), respectively). In children aged ≤9 years at study end, axial length at study end was 0.20 mm larger than axial length at baseline in the participants aged ≤9 years at baseline. Larger axial elongation during the study period was associated (multivariable analysis) with younger age (beta:-0.62;*P*<0.001), female sex (beta:0.21;*P*<0.001), longer study period (beta:0.22;*P*<0.001), and longer axial length at baseline (beta:0.28;*P*<0.001), and marginally, with less time spent outdoors (beta:-0.07;*P* = 0.06).

**Conclusions:**

The COVID-19-related lockdown in the Russian city of Ufa was associated with a relatively minor increase in axial elongation, detected only in children aged <9.6 years.

## Introduction

The prevalence of axial myopia has markedly increased during the last decades, in particular in East and Southeast Asia [1, 2]. Pathologic myopia has been reported to be one of the most common causes of irreversible vision impairment and blindness [3]. Numerous studies have convincingly shown that higher prevalence and incidence of myopia in children and adolescents is associated with more time spending indoors and less time spending outdoors [4–11]. In a parallel manner, adult Chinese born in the period from 1958 to 1961 and who were prevented from attending the first school classes in the early years of Cultural Revolution in China are less myopic than Chinese born earlier or later [12]. Correspondingly, one has discussed that the restrictions due to the public lockdown in association with the COVID-19 (coronavirus disease 2019) pandemic may also have an effect of myopization on children and adolescents [13–21]. In a recent study on almost 200,000 school children in Feicheng in the Chinese province of Shandong, Wang and colleagues found that children aged 6 to 8 years had a lockdown-associated increase in myopia by about -0.3 diopters [15]. In contrast, children aged 9 to 13 years showed only minor differences in refractive error and prevalence of myopia between 2020 and the previous years. Chang and colleagues examined 29,719 out of 44,187 (67.3%) children in a longitudinal manner and reported about accelerated myopic progression in association with the COVID-19-related lockdown [16]. These studies had some limitations, in particular the use of non-cycloplegic photoscreening measurement of the refractive error, missing information on previous or current procedures to reduce myopia progression like orthokeratology or topical low-dose atropine therapy, lack of ocular biometric data, and being confined to Chinese children. We, therefore, conducted the present study to assess in a multiethnic school children population in Russia the effect of a COVID-19 associated lockdown on the development of myopic refractive error in children without any preventive therapy of myopia progression, and applying biometry for measurement of axial length, and refractometry for the measurement of the refractive error.

## Materials and methods

The Ural Children Myopia Study included children from four randomly selected schools located in various regions of the Kirovskii district in the city of Ufa. The Ethics Committee of the Academic Council of the Ufa Eye Research Institute approved the study design and confirmed that the study adhered to the Declaration of Helsinki, and at least one the parents gave an informed written consent. Ufa is the capital of the republic of Bashkortostan / Russia and is an industrial, economic, scientific and cultural center. With altogether 1.1 million inhabitants, the citizenship of Ufa is ethnically composed of Russians, Tatars, Bashkirs, and other ethnicities. The republic of Bashkortostan, with a population of approximately 4.07 million inhabitants, is situated at the southwestern end of the Ural Mountains. The Kirovskii district is one of seven urban districts of Ufa, is located in the southern part of Ufa, and includes 165,000 inhabitants of various ethnically background. The district is the business, cultural, and scientific center of the capital of Bashkortostan. There are 20 large and medium-sized industrial enterprises that produce medical products, electrical equipment, communication equipment, clothing and textiles, food, and other products. The Kirovskii district includes 18 schools with children of grade 1 to 11. Out of these 18 schools, we randomly selected four schools (total number of pupils per school: 598, 1030, 2099,1936). Inclusion criterion for the participation in the study was attending one of the four randomly selected schools at the grades of 1 to grades 11. Exclusion criteria were the use of topical low-dose atropine eye drops or orthokeratology as another procedure to reduce the progression of myopia. At the time of study, low-concentration atropine eye drops, orthokeratology or other measures to prevent further myopia progression had only rarely been applied in the study region.

All children of the Ural Children Study came to the Ufa Eye Research Institute for the examinations. The children and their parents underwent a standardized interview performed by trained social workers who personally asked the questions and filled the answers into datafile sheets. The questionnaires included questions on diet (vegetarian versus mixed diet, number of daily meals, frequency and amount of intake of vegetables, fruits and meat, type of cooking oil used, intake of food with whole grain, estimated salt consumption, degree of meat processing), daily physical activity (walking, cycling or going by bus to school, time of running or walking per day, time spent with playing sport games (basketball, volleyball, badminton, football), cognitive function, presence of any specific ocular problems or disorders, hereditary eye diseases, availability and wearing of glasses, sunglasses and medical history including known diagnosis and therapy of major diseases such as arterial hypertension, diabetes mellitus, and previous trauma including bone fractures. The questions were taken from standardized questionnaires, such as the Mini-Mental Status Examination test for the assessment of cognitive function [22]. Additional COVID-19 pandemic-associated questions were taken from the “Convergence Insufficiency Symptom Survey”, the “Computer Vision Syndrome Assessment”, the “Computer Activities and Environment Assessment”, the “Computer Vision Syndrome Assessment”, the “General Health Questionnaire (GHQ) 12” and the “Generalized Anxiety Disorder Scale-7” (GAD-7) for the assessment of depression and anxiety [23–27].

The non-ophthalmological examinations consisted of measurement of the anthropometric parameters of body height and body weight and measurement of the hand grip force by dynamometry. The ophthalmological examinations included testing of presenting visual acuity (with habitual glasses), uncorrected visual acuity and best-corrected visual acuity by ophthalmologists. We used the modified Early Treatment of Diabetic Retinopathy Study (ETDRS) charts (Light House Low Vision Products, New York, NY) at a distance of 4 meters. Best-corrected visual acuity was measured based on the results of automated refractometry (Auto Ref/Kerato/Tono/Pachymeter Tonoref, Nidek, Japan), and subsequent subjective refractometry. Refractometric readings were obtained under relative cycloplegic conditions, after applying tropicamide 0,8% eyes drops once (Mydrimax®; Sentiss Co., Gurugram, Haryana 122001, India). Additional examinations were Schober’s test to assess heterophorias, assessment of corneal hysteresis and corneal resistance using the Ocular Response Analyzer (ORA, Reichert, Inc., USA) and the Corvis ST device (Oculus Inc, Germany), tonometry, imaging of the anterior ocular using a Scheimflug camera (Pentacam HR, Typ70900, OCULUS, Optikgeräte GmbH Co., Wetzlar, Germany), slit lamp-based biomicroscopy carried out by a fellowship-trained ophthalmologist, and laser interferometric biometry (AL-Scan, Nidek Co, Ltd, Gamagory, Japan). Additionally, we took digital photographs of the cornea and lens (Topcon slit lamp and camera, Topcon Corp. Tokyo, Japan) and of the optic nerve head and macular (60° images; VISUCAM 500, Carl Zeiss Meditec AG, Jena, Germany), and we performed a swept-source optical coherence tomography (OCT) (swept-source OCT Triton, Topcon Corporation, Itabashi-ku, Tokyo 174 8580, Japan) with images taken from the macula and optic nerve head.

The examinations were performed at baseline in the period from January 2019 to March 2020 before the initiation of a pandemic-related lockdown in Bashkortostan, which started on March 18^th^, 2020 and lasted till June 2020. During this lockdown, the children were not allowed to go outside, play outdoors, or go to public places. The regulations of the lockdown were controlled by the police. The schools were closed till December 7^th^, 2020. After the end of the public lockdown in June 2020, when the schools were still closed, the children were allowed to be outdoors, but they spent most of their time indoors because of distance school learning. Sports activities were allowed from September 2020 onwards. If possible, the parents sent their children in the summer vacations outside of the city to grandparents in the countryside. The children were re-examined about one to two years after the baseline examination.

The statistical analysis was performed using a statistical package analysis program (SPSS for Windows, version 27.0, IBM-SPSS, Chicago, IL, USA). In a first step, we assessed, in the total study population and in subgroups stratified by age, the mean and standard deviation of axial length at baseline before the outbreak of the COVID-19 pandemic and at study end. Performing a linear regression analysis, we determined the relationship between axial length at baseline and age at baseline and used the steepness of the regression line to determine the mean increase in axial length per year of age before the COVID-19 outbreak. This value was compared with the amount of the annual axial elongation during the study period, determined as the difference in axial length between study end and baseline and divided by the number of years of the duration of the study period. In a second step, we compared the mean axial length in age-defined groups of participants before the COVID-19 outbreak and at study end. Using linear regression analyses, we finally assessed associations between the axial elongation during the study period and ocular and systemic parameters, first in univariable analyses and followed by a multivariable analysis. We calculated the standardized regression coefficient beta, the non-standardized regression coefficient B and its 95% confidence intervals (CIs). All *P*-values were two-sided and considered statistically significant when the values were less than 0.05.

## Results

The study included 461 school children (235 (50.1%) boys, 234 (49.9%) girls) with a mean age of 10.7 ± 2.1 years (median: 10.6 years; range: 6.8–16.9 years) at baseline and of 12.2 ± 2.2 years (median: 12.0 years; range: 7.8–18.8 years) at study end. The duration of the follow-up was 1.41 ± 0.33 years (median: 1.41 years; range: 0.73–1.98 years).

**Fig 1:**
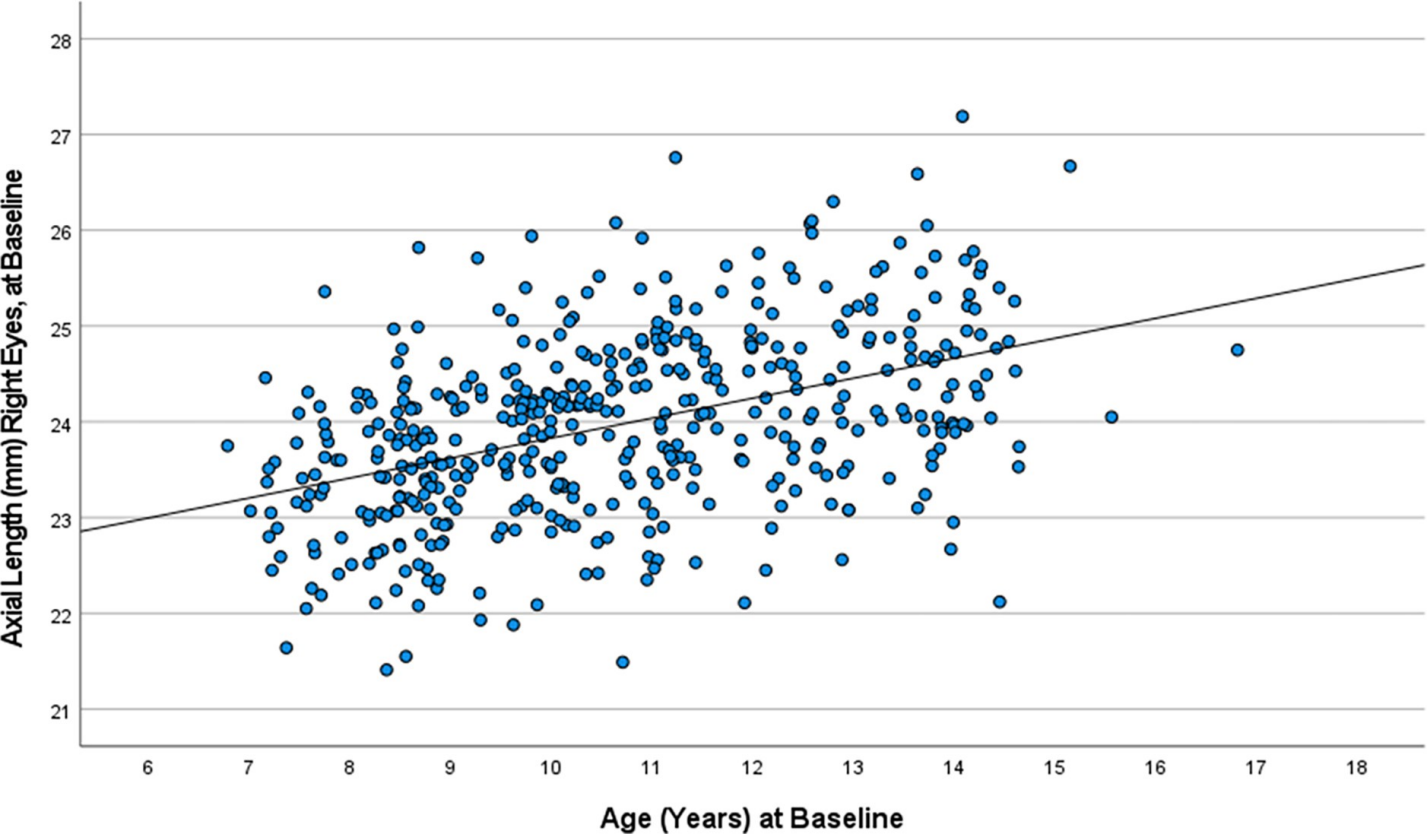
Scattergram showing the distribution of axial length in the right eyes before start of the COVID-19 lockdown, stratified by age in the Ural Children Eye Study. Equation of the regression line: Axial Length (mm) = 21.74 mm + 0.21 (95%CI: 0.17, 0.25) x Age (Years).

Mean axial length measured at baseline was 23.96 ± 0.95 mm in the right eyes and 23.94 ± 0.95 mm in the left eyes (with no significant difference between both eyes (*P* = 0.09)). Both parameters increased significantly (both *P*<0.001; right eyes, r^2^ = 0.20; left eyes, r^2^ = 0.19) with older age (equation of the regression line, right eyes: Axial Length (mm) = 21.74 mm + 0.21 (95%CI: 0.17, 0.25) x Age (Years); left eyes: Axial Length (mm) = 21.81 mm + 0.20 (95%CI: 0.16, 0.24) x Age (Years)) (Fig 1). Taking into account the steepness of the regression lines, the age-dependent coefficient for axial length (ADCAL) at baseline of the study was 0.21 mm (95%CI: 0.17, 0.25) for the right eyes and 0.20 mm (95%CI: 0.16, 0.24) for the left eyes. After adjusting for age, the mean axial length was 23.98 ± 0.85 mm in the right eyes and 23.95 ± 0.85 mm in the left eyes at baseline.

**Fig 2:**
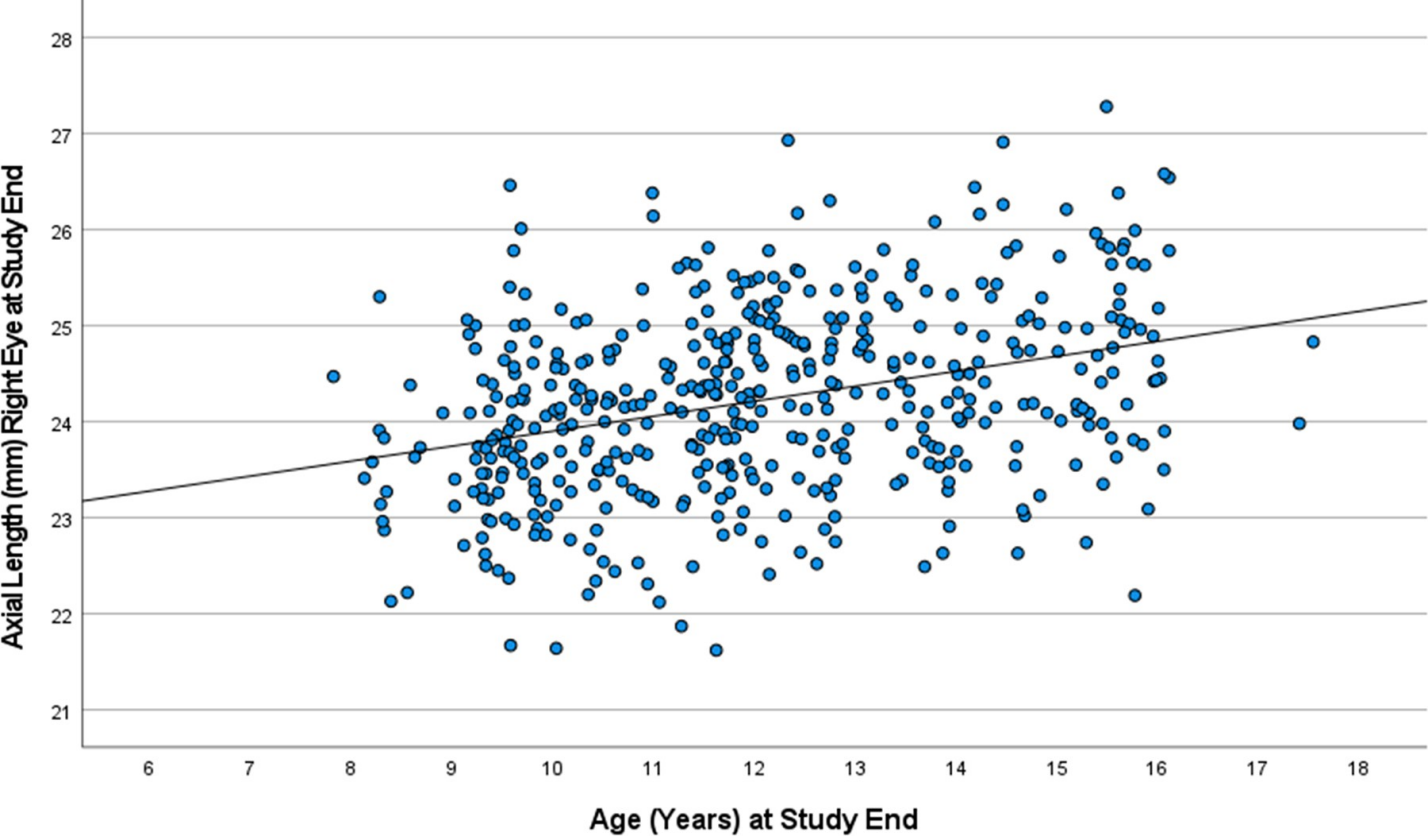
Scattergram showing the distribution of axial length in the right eyes at study end, stratified by age in the Ural Children Eye Study. Equation of the regression line: Axial Length (mm) = 22.34 mm + 0.16 (95%CI: 0.12, 0.20) x Age (Years).

Mean axial length measured at study end was 24.22 ± 0.97 mm in the right eyes and 24.20 ± 0.99 mm in the left eyes (with no significant difference between both eyes (*P* = 0.24)), with a significant (both *P*<0.001) increase during the study period of 0.26 ± 0.23 mm in the right eyes and of 0.26 ± 0.29 mm in the left eyes. The annual axial elongation during the study period was 0.19 ± 0.17 mm for the right eyes and 0.19 ± 0.22 mm for the left eyes. The axial length of both eyes increased significantly (both *P*<0.001; right eyes, r^2^ = 0.11; left eyes, r^2^ = 0.11) with older age (equation of the regression line, right eyes: Axial Length (mm) = 22.34 mm + 0.16 (95%CI: 0.12, 0.20) x Age (Years); left eyes: Axial Length (mm) = 22.32 mm + 0.16 (95%CI: 0.12, 0.20) x Age (Years)) (Fig 2). After adjusting for age, the mean axial length was 24.24 ± 0.92 mm in the right eyes and 24.22 ± 0.94 mm in the left eyes at study end.

Mean refractive error at baseline was -1.59 ± 1.48 diopters (D) in the right eyes and -1.54 ± 1.44 D in the left eyes, with no significant difference between both eyes (*P* = 0.10). The myopic refractive error of both eyes increased significantly (both *P*<0.001; right eyes, r^2^ = 0.13; left eyes, r^2^ = 0.10) with older age (equation of the regression line, right eyes: Refractive Error (D) = 1.10–0.25 (95%CI: -0.32, -0.19) x Age (Years); left eyes: Refractive Error (D) = 0.85–0.23 (95%CI: -0.29, -0.16) x Age (Years)). Taking into account the steepness of the regression lines, the mean increase in myopic refractive error for each year of age at baseline of the study was -0.25 D (95%CI: -0.32, -0.19) for the right eyes and -0.23 D (95%CI: -0.29, -0.16) for the left eyes. After adjusting for age, the mean refractive error was -1.61 ± 1.35 D in the right eyes and -1.77 ± 1.64 D in the left eyes at baseline.

Mean refractive error measured at study end was -1.74 ± 1.71 D in the right eyes and -1.72 ± 1.71 D in the left eyes, with no significant difference between both eyes (*P* = 0.62), with a significant (both *P*<0.001) increase in myopic refractive error during the study period of -0.12 ± 0.84 D in the right eyes and of -0.16 ± 0.79 D in the left eyes. The increase in refractive error of the right eyes increased marginally significantly with older age at baseline (*P* = 0.01; r^2^ = 0.01) (equation of the regression line, right eyes: Refractive Error (D) = -0.63 + 0.05 (95%CI: 0.01, 0.09) x Age (Years), while the change in refractive error during the study period in the left eyes was not significantly associated with ag eat baseline *(P =* 0.07; r^2^ = 0.01).

Comparison of the ADCAL at baseline and the annual axial elongation during the study period in the total study population, the annual axial elongation during the study period was similar or slightly smaller than the ADCAL before the COVID-19 outbreak (0.19 mm (95%CI: -0.09, 0.59) versus 0.21 mm (95%CI: 0.17, 0.25) for the right eyes; 0.19 mm (95%CI: -0.09, 0.58) versus 0.20 mm (95%CI: 0.16, 0.24) for the left eyes) (Table 1).

**Table 1:** Axial length (mean ± standard deviation) in the Ufa Children Myopia Study.

Age (Years) at Baseline	n	Axial length (mm) (right eye), baseline	Axial length (mm) (right eye), study end	Annual axial elongation (mm) (right eyes) during the Corona period	Annual axial elongation (mm) (right eyes) before the Corona period	Axial length (mm) (left eye), baseline	Axial length (mm) (left eye), study end	Annual axial elongation (mm) (left eyes) during the Corona period	Annual axial elongation (mm) (left eyes) before the Corona period
Total	461	23.96 ± 0.95	24.22 ± 0.97	0.19 ± 0.17	0.21 (95%CI: 0.17, 0.25)	23.94 ± 0.95	24.20 ± 0.99	0.19 ± 0.22	0.20 (95%CI: 0.16, 0.24)
<9.6 years	157	23.44 ± 0.78	23.82 ± 0.88	0.29 ± 0.17	0.21 (95%CI: 0.02, 0.41)	23.43 ± 0.79	23.80 ± 0.88	0.28 ± 0.17	0.21 (95%CI: 0.02, 0.41)
9.6 to 11.4	148	24.03 ± 0.87	24.28 ± 0.93	0.18 ±0.15	0.22 (95%CI: -0.05, 0.48)	24.01 ± 0.87	24.24 ± 0.93	0.16 ± 0.16	0.17 ((95%CI: -0.09, 0.44)
>11.4	156	24.43 ± 0.92	24.57 ± 0.96	0.09 ± 0.12	0.14 (95%CI: 0.00, 0.28)	24.40 ± 0.91	24.57 ± 1.02	0.12 ± 0.28	0.12 (95%CI: -0.03, 0.26)

To analyze a potential effect of the lockdown on axial length in dependence of age, we divided the study population into three, mostly equally sized groups. In the group of study participants younger than 9.6 years (n = 157 children), annual axial elongation in the right eyes during the study period was larger than the ADCAL before the COVID-19 outbreak (0.29 mm (95%: 0.00, 0.66) versus 0.21 mm (95%CI: 0.02, 0.41)) (Table 1). In the group aged 9.6 to 11.4 years (n = 148 children), the annual axial elongation in the right eyes during the study period was comparable to (slightly smaller than) the ADCAL before the COVID-19 outbreak (0.18 mm (95%CI: -0.07, 0.46) versus 0.22 mm (95%CI: -0.05, 0.48)). In a similar manner in the group aged >11.4 years (n = 156 individuals), the annual axial elongation in the right eyes during the study period was comparable to (slightly smaller than) the ADCAL before the COVID-19 outbreak (0.09 mm (95%CI: -0.15, 0.34) versus 0.14 mm (95%CI: 0.00, 0.28)) (Table 1).

In the participants aged ≤9 years at study end, the axial length at study end was larger by 0.20 mm than the axial length at baseline in the participants aged ≤9 years at baseline (Table 2). For the other age groups, the difference ranged between -0.08 mm in the group aged >10 to 11 years (i.e., axial length was shorter in the cohort aged at >10 to 11 years at study end than in the cohort aged >10 to 11 years at baseline) and +0.11 mm in the group aged >12 to 13 years (Table 2).

**Table 2:** Axial length (mean ± standard deviation) in the Ufa Children Myopia Study, stratified by age at baseline and at the end of the study period.

Age (years) at Baseline	n	Axial length (mm) (right eye), baseline	Age (years) at study end	n	Axial length (mm) (right eye), study end	Axial length difference (mm) (study end minus baseline)
≤9	127	23.36 ± 0.77	≤9	16	23.56 ± 0.82	0.20
>9 to 10	67	23.87 ± 0.78	>9 to 10	77	23.82 ± 0.89	-0.05
>10 to 11	76	23.98 ± 0.89	>10 to 11	71	23.90 ± 0.88	-0.08
>11 to 12	64	24.22 ± 0.85	>11 to 12	83	24.18 ± 0.88	-0.04
>12 to 13	52	24.30 ± 0.96	>12 to 13	69	24.41 ± 0.97	0.11
>13	75	24.61 ± 0.91	>13	145	24.60 ± 0.96	-0.01

In univariate analysis, axial elongation during the study period was associated with several systemic and ocular parameters (Table 3). In the multivariable analysis, we dropped parameters due to collinearity and due to a lack of statistical significance. In the final model, a larger axial elongation (right eyes) during the study period was associated with younger age (beta: -0.62; B: -0.07; 95%CI: -0.08, -0.06; *P*<0.001), female sex (beta: 0.21; B: 0.10; 95%CI: 0.06, 0.13; *P*<0.001), longer study period (beta: 0.22; B: 0.16; 95%CI: 0.10, 0.21; *P*<0.001), and longer axial length at baseline (beta: 0.28; B: 0.07; 95%CI: 0.05, 0.09; *P*<0.001). Similar findings were made for the axial elongation of the left eyes (younger age (beta: -0.45; B: -0.06; 95%CI: -0.08, -0.05; *P*<0.0 dominant hand (*P* = 0.96), dominant hand (*P* = 0.96), 01), female sex (beta: 0.18; B: 0.10; 95%CI: 0.05, 0.15; *P*<0.001), longer study period (beta: 0.13; B: 0.11; 95%CI: 0.04, 0.19; *P* = 0.003), and longer axial length at baseline (beta: 0.23; B: 0.07; 95%CI: 0.04, 0.10; *P*<0.001). In that model, less time spent outdoors for more marked axial elongation had a marginal significant effect (beta: -0.07; B: -0.02; 95%CI: -0.04, 0.00; *P* = 0.06).

**Table 3:** Associations (univariable analysis) between the axial elongation during the study period and systemic and ocular parameters.

Parameter	Standardized regression coefficient beta	P-value
Age (years)	-0.46	<0.001
Gender (boys / girls)	0.12	0.01
Length of follow-up period	0.17	<0.001
School location	0.12	0.009
Body height	-0.44	<0.001
Body weight	-0.35	0.001
Body mass index	-0.18	<0.001
Systolic blood pressure	-0.17	<0.001
Mini-mental test score	-0.23	<0.001
Number of daily meals	0.12	0.01
Number of days with fruit intake	-0.14	0.003
Number of days with vegetable intake	-0.15	0.001
Daily salt consumption	-0.15	0.002
Smoking of the father before pregnancy	0.12	0.01
Smoking of the father during pregnancy	0.11	0.02
Smoking of the father after pregnancy	0.12	0.01
Time spending outdoors before leaving to school	-0.10	0.03
Time spent outdoors during the weekend	-0.12	0.01
Daily sleep duration	0.10	0.03
Paternal degree of myopia	0.13	0.007
Maternal degree of myopia	-0.15	0.001
Diastolic blood pressure,		0.83
School type		0.92
School marks in languages		0.16
School marks in mathematics		0.06
School marks in science		0.16
School marks in social science		0.19
Dominant hand		0.96
Type of cooking oil used		0.59
Intake of food rich in whole grains		0.52
Degree of meat processing		0.61
Number of cups of coffee taken daily,		0.14
Number of cups of tea taken daily,		0.40
Ethnicity (Russian versus non-Russian)		0.26
Vacation outside of the home		0.72
Possession of second house		0.22
Possession of a laptop or personal computer		0.70
Possession of a second car		0.37
Type of returning from school, walking:		0.54
Type of returning from school, school bus:		0.32
Paternal ethnic background		0.65
Maternal ethnic background		0.75
Paternal body height		0.56
Maternal body height		0.94
Paternal body weight		0.35
Maternal body weight		0.93
Paternal occupation		0.43
Maternal occupation		0.31
Paternal level of education		0.56
Maternal level of education		0.91
Maternal age at birth		0.41
Paternal marital status		0.23
Maternal marital status		0.33
Paternal ethnicity		0.25
Maternal ethnicity		0.14
Smoking of the mother before pregnancy		0.06
Smoking of the mother during pregnancy		0.63
Smoking of the mother after pregnancy		0.66
Alcohol consumption of the father before pregnancy		0.85
Alcohol consumption of the father during pregnancy		0.80
Alcohol consumption of the father after pregnancy		0.55
Alcohol consumption of the mother before pregnancy		0.29
Alcohol consumption of the mother during pregnancy		0.25
Alcohol consumption of the mother after pregnancy		0.32
Preference of sweet food		0.41
Time spent on the way back from school		0.62
Axial length at baseline		0.60
Intraocular pressure		0.16
Central corneal thickness		0.07
Anterior corneal curvature radius		0.054
Wearing sun glasses		0.40

## Discussion

This school-based study included school children aged 7 and 19 years, who been examined before the start of the COVID-19-related lockdown and who had been re-examined after the end of the home-based school distance learning of 8 months and after a total mean follow-up of 1.41 ± 0.33 years. The study revealed only a relatively minor effect of the home-schooling on axial elongation. Only in the group of study participants younger than 9.6 years, the annual axial elongation during the study period was larger than the mean annual increase in axial length at baseline (0.29 mm versus 0.21 mm) (Table 1). In the other groups aged 9.6 to 11.4 years and aged >11.4 years the annual axial elongation during the study period was comparable to or slightly smaller than the mean annual increase in axial length at baseline (0.18 mm versus 0.22 mm, and 0.09 mm versus 0.14 mm, respectively) (Table 1). Correspondingly, in the children aged ≤9 years at study end, the axial length at study end was larger by 0.20 mm than the axial length at baseline in the participants aged ≤9 years at baseline (Table 2). For the other age groups, the differences were smaller or even negative. Subsequently, a larger axial elongation during the study period was associated with younger age, in addition to female sex, longer study period, and longer axial length at baseline, and marginally, with less time spent outdoors.

These findings agree with observations reported by Wang and colleagues from China, who measured the refractive error of close to 200,000 school children by non-cycloplegic photoscreening and reported on a myopic shift of approximately -0.3 diopters occurring in association with the pandemic-related home confinement in children aged 6 to 8 years [15]. In children aged 9 to 13 years, the differences in the myopic refractive error between 2020 and years before the COVID-19 associated lockdown were minor. With respect to Wang´s study, it may be taken into account that the 6-year-olds, for whom Wang and colleagues reported on to show a COVID effect, were preschool children who had not yet started school; that they were tested early, and that a possible further development of myopia over the three months until the children started school may suggest that the change was under-estimated. The results of our study also agree with the findings obtained in the investigation by Chang and colleagues [16]. Similar observations were made in other investigations [28–33]. The results of our study as well as the findings obtained in the studies by Wang and Chang and colleagues agree with the results of the Beijing Eye Study, which, in a reverse manner, found a less myopic refractive error for study participants who had an age of 6 to seven years when they should have gone to school however were blocked due to the closure of the schools at the beginning of China´s “Great Leap Forward” (“cultural revolution”) and could not start studying [12]. School closure in those days was not combined with home-confinement. The results of Wang´s and Chang´s investigation and others our study also agree with a trial in which adding a 40-minute class of outdoor activities and encouragement of spending more time outdoors was associated with a significant decrease in the incidence of myopia in school children in Guangzhou/China [9]. It is also in agreement with numerous studies showing in cross-sectional and longitudinal analyses an association between less time spent outdoors and a higher prevalence and incidence of myopia in school-aged children [5–7, 10, 11].

It has remained undetermined why children younger than 9.6 years as compared to older children in our study population showed a stronger association with the COVID>-lockdown, i.e., why they may have been predisposed to increased myopia, especially with a reduced exposure to outdoor light during the lockdown period. With the same observation made in investigations of study populations of other ethnicities and countries, one may assume that younger children are more sensitive to the environmental change than older children [15].

In the discussion about negative effects of an increased myopic shift in children and adolescents, such as an increased risk for rhegmatogenous retinal detachment and the need for myopia correction by glasses or other optical measures, one may not overlook some advantages of axial, minor to moderate, myopia, such as a decreased risk for age-related macular degeneration and diabetic retinopathy as well as, in the case of minor to moderate myopia, the possibility of unaided good near vision in the presbyopic age period [34, 35].

When the results of our study are discussed, its limitations have to be taken into account. First, it was performed in a city of one million inhabitants in Russia in an area with otherwise low population density, where the parents had ample possibilities to spend their weekends and vacations in the countryside. The results may, therefore, not be fully comparable to studies performed in megacities. Second, it was not possible to have a control group of different individuals, so that we compared, within the same study population, axial length in relationship to time only between the period before the lockdown and the period after the lockdown. As a comparison of slopes, this approach may not give easily interpretable findings. Third, another weakness of the study design is that the conclusion was based on comparing a longitudinal within-person change to a cross-sectional, between-person difference. Although they may be similar, there may not be a full assurance that these two estimates are comparable. Fourth, we performed a linear regression analysis between axial length and age, although it may not be clear that a linear fit the best. Axial elongation slows with older age in school children so that the association between axial elongation and age may be non-linear. Fifth, the lockdown period was relatively short with about 3.5 months. The follow-up period ranged from 0.73 to 1.98 years. On the basis of the two measurements obtained at baseline of the study and its end, the effect of a short-term change (3.3 month) in external factors (lockdown) at a random period between the measurements, varying 14% to 40% of the follow-up, may be difficult to be reliably calculated. Sixth, the interpretation of the results of this study is influenced by the fact that the change in axial length is not linear and varies depending on several parameters. The large-scale longitudinal CLEERE study found that children aged 6 to 14 who remained emmetropic showed an average of 0.1 mm per year axial growth [8]. More specifically by age, this was 0.16 mm per year for age 6–9 years, 0.08 mm per year for age 9–12 and 0.02 mm annually for age group 11–14 years. Thereafter axial length among the non-myopics did practically not increase any more. In myopic children, the increase of myopia (axial length) was generally faster at the beginning, and thereafter slowed down. In general, there was a marked inter-individual variability. These findings show that it may be difficult to compare the increase in axial length before a timepoint (in our study, the beginning of the study period with a lock-down phase) and after a timepoint. Sixth, since we applied only tropicamide eyes drops for cycloplegia, the latter was probably incomplete. Strengths of our study were that axial length was the primary outcome parameter, and that the influence of procedures to decrease myopia progression, such as topical low-dose atropine therapy or orthokeratology were exclusion criteria.

In conclusion, the COVID-19-related lockdown in the Russian city of Ufa was associated with a relatively minor increase in axial elongation, detected only in children aged <9.6 years.

## Supporting information

S1 File (DOCX)

S2 File (SAV)

## References

[1] Morgan IG, Ohno-Matsui K, Saw SM. Myopia. Lancet. 2012;379(9827):1739–1748. doi: 10.1016/S0140-6736(12)60272-4 22559900

[2] Dong L, Kang YK, Li Y, Wei WB, Jonas JB. Prevalence and time trends of myopia in children and adolescents in China: a systemic review and meta-analysis. Retina. 2020;40(3):399–411. doi: 10.1097/IAE.0000000000002590 31259808

[3] Xu L, Wang Y, Li Y, Li J, Wang Y, Cui T, et al. Causes of blindness and visual impairment in urban and rural areas in Beijing: the Beijing Eye Study. Ophthalmology. 2006;113(7):1134–1141. doi: 10.1016/j.ophtha.2006.01.035 16647133

[4] Jones LA, Sinnott LT, Mutti DO, Mitchell GL, Moeschberger ML, Zadnik K. Parental history of myopia, sports and outdoor activities, and future myopia. Invest Ophthalmol Vis Sci. 2007;48(8):3524–3532. doi: 10.1167/iovs.06-1118 17652719 PMC2871403

[5] Rose KA, Morgan IG, Ip J, Kifley A, Huynh S, Smith W, et al. Outdoor activity reduces the prevalence of myopia in children. Ophthalmology. 2008;115(8):1279–1285. doi: 10.1016/j.ophtha.2007.12.019 18294691

[6] Guo Y, Liu LJ, Xu L, Lv YY, Tang P, Feng Y, et al. Outdoor activity and myopia among primary students in rural and urban regions of Beijing. Ophthalmology. 2013;120(2): 277–283. doi: 10.1016/j.ophtha.2012.07.086 23098368

[7] Wu LJ, Wang YX, You QS, Duan JL, Luo YX, Liu LJ, et al. Risk factors of myopic shift among primary school children in Beijing, China: a prospective study. Int J Med Sci. 2015;12(8):633–638. doi: 10.7150/ijms.12133 26283882 PMC4532970

[8] Zadnik K, Sinnott LT, Cotter SA, Jones-Jordan LA, Kleinstein RN, Manny RE, et al. Prediction of juvenile-onset myopia. JAMA Ophthalmol. 2015;133(6):683–689. doi: 10.1001/jamaophthalmol.2015.0471 25837970 PMC4607030

[9] He M, Xiang F, Zeng Y, Mai J, Chen Q, Zhang J, et al (2015). Effect of time spent outdoors at school on the development of myopia among children in China: a randomized clinical trial. JAMA. 314(11):1142–1148. doi: 10.1001/jama.2015.10803 26372583

[10] Pärssinen O, Kauppinen M. Risk factors for high myopia: a 22-year follow-up study from childhood to adulthood. Acta Ophthalmol. 2019;97(5):510–518. doi: 10.1111/aos.13964 30460746

[11] Yotsukura E, Torii H, Inokuchi M, Tokumura M, Uchino M, Nakamura K, et al. Current prevalence of myopia and association of myopia with environmental factors among schoolchildren in Japan. JAMA Ophthalmol. 2019;137(11):1233–1239. doi: 10.1001/jamaophthalmol.2019.3103 31415060 PMC6696729

[12] Wang YX, Xu L, Jonas JB. The effect of the Chinese Cultural Revolution and Great Leap Forward on the prevalence of myopia. Eur J Epidemiol. 2013;28(12):1001–1004. doi: 10.1007/s10654-013-9858-z 24197389

[13] Wang G, Zhang Y, Zhao J, Zhang J, Jiang F. Mitigate the effects of home confinement on children during the COVID-19 outbreak. Lancet. 2020;395(10228):945–947. doi: 10.1016/S0140-6736(20)30547-X 32145186 PMC7124694

[14] Wong CW, Tsai A, Jonas JB, Ohno-Matsui K, Chen J, Ang M, et al. Digital screen time during the COVID-19 pandemic: risk for a further myopia boom? Am J Ophthalmol. 2021;223:333–337. doi: 10.1016/j.ajo.2020.07.034 32738229 PMC7390728

[15] Wang J, Li Y, Musch DC, Wei N, Qi X, Ding G, et al. Progression of myopia in school-aged children after COVID-19 home confinement. JAMA Ophthalmol. 2021;139(3):293–300. doi: 10.1001/jamaophthalmol.2020.6239 33443542 PMC7809617

[16] Chang P, Zhang B, Lin L, Chen R, Chen S, Zhao Y, et al. Comparison of the myopic progression before, during and after COVID-19 lockdown. Ophthalmology. 2021;128(11):1655–1657. 33771516 10.1016/j.ophtha.2021.03.029 PMC7986471

[17] Ma M, Xiong S, Zhao S, Zheng Z, Sun T, Li C. COVID-19 home quarantine accelerated the progression of myopia in children aged 7 to 12 years in China. Invest Ophthalmol Vis Sci. 2021;62(10):37. doi: 10.1167/iovs.62.10.37 34463719 PMC8411864

[18] Alvarez-Peregrina C, Martinez-Perez C, Villa-Collar C, Andreu-Vázquez C, Ruiz-Pomeda A, Sánchez-Tena MÁ. Impact of COVID-19 home confinement in children’s refractive errors. Int J Environ Res Public Health. 2021;18(10):5347. doi: 10.3390/ijerph18105347 34067888 PMC8156137

[19] Yang X, Fan Q, Zhang Y, Chen X, Jiang Y, Zou H, et al. Changes in refractive error under COVID-19: a 3-year follow-up study. Adv Ther. 2022;39(6):2999–3010. doi: 10.1007/s12325-022-02150-0 35508845 PMC9067555

[20] Mu J, Zhong H, Liu M, Jiang M, Shuai X, Chen Y, et al. Trends in myopia development among primary and secondary school students during the COVID-19 pandemic: a large-scale cross-sectional study. Front Public Health. 2022;10:859285. doi: 10.3389/fpubh.2022.859285 35392469 PMC8980682

[21] Cai T, Zhao L, Kong L, Du X. Complex interplay between COVID-19 lockdown and myopic progression. Front Med (Lausanne). 2022;9:853293. doi: 10.3389/fmed.2022.853293 35386915 PMC8978626

[22] Folstein MF, Folstein SE, McHugh PR. "Mini-mental state". A practical method for grading the cognitive state of patients for the clinician. J Psychiatr Res. 1975;12(3):189–198. doi: 10.1016/0022-3956(75)90026-6 1202204

[23] Cao W, Fang Z, Hou G, Han M, Xu X, Dong J, et al. The psychological impact of the COVID-19 epidemic on college students in China. Psychiatry Res. 2020;287:112934. doi: 10.1016/j.psychres.2020.112934 32229390 PMC7102633

[24] Clark TY, Clark RA. Convergence Insufficiency Symptom Survey scores for reading versus other near visual activities in school-age children. Am J Ophthalmol. 2015;160(5):905–912. doi: 10.1016/j.ajo.2015.08.008 26275474

[25] Liang L, Ren H, Cao R, Hu Y, Qin Z, Li C, et al. The Effect of COVID-19 on Youth Mental Health. Psychiatr Q. 2020;91(3):841–852. doi: 10.1007/s11126-020-09744-3 32319041 PMC7173777

[26] Mar Segui M, Cabrero-García J, Crespo A, Verdú J, Ronda E. A reliable and valid questionnaire was developed to measure computer vision syndrome at the workplace. J Clin Epidemiol. 2015;68(6):662–673. doi: 10.1016/j.jclinepi.2015.01.015 25744132

[27] Pierce M, Hope H, Ford T, Hatch S, Hotopf M, John A, et al. Mental health before and during the COVID-19 pandemic: a longitudinal probability sample survey of the UK population. Lancet Psychiatry. 2020;7(10):883–892. doi: 10.1016/S2215-0366(20)30308-4 32707037 PMC7373389

[28] Chen H, Liao Y, Zhou W, Dong L, Wang W, Wang X. The change of myopic prevalence in children and adolescents before and after COVID-19 pandemic in Suqian, China. PLoS One. 2022;17(3):e0262166. doi: 10.1371/journal.pone.0262166 35312694 PMC8937315

[29] Ma D, Wei S, Li SM, Yang X, Cao K, Hu J, et al. The impact of study-at-home during the COVID-19 pandemic on myopia progression in Chinese children. Front Public Health. 2022;9(: 720514. doi: 10.3389/fpubh.2021.720514 35071149 PMC8770940

[30] Wang W, Zhu L, Zheng S, Ji Y, Xiang Y, Lv B, et al. Survey on the progression of myopia in children and adolescents in Chongqing during COVID-19 pandemic. Front Public Health. 2021;9:646770. doi: 10.3389/fpubh.2021.646770 33996724 PMC8115404

[31] Yang YC, Hsu NW, Wang CY, Shyong MP, Tsai DC. Prevalence trend of myopia after promoting eyecare in preschoolers: a serial survey in Taiwan before and during the COVID-19 pandemic. Ophthalmology. 2021;129(2):181–190. 34425129 10.1016/j.ophtha.2021.08.013

[32] YaoY. FuJ, LiuJ, LiL, ChenW, MengZ, DaiW. Distribution, progression, and associated factors of refractive status of children in Lhasa, Tibet after COVID-19 quarantine. Ophthalmic Res. 2022;65(3):321–327. doi: 10.1159/000522548 35172321

[33] Zhang X, Cheung SSL, Chan HN, Zhang Y, Wang YM, Yip BH, et al. Myopia incidence and lifestyle changes among school children during the COVID-19 pandemic: a population-based prospective study. Br J Ophthalmol. 2021 Aug 2;bjophthalmol-2021-319307. Online ahead of print. doi: 10.1136/bjophthalmol-2021-319307 34340973

[34] Xu L, Li Y, Zheng Y, Jonas JB. Associated factors for age-related maculopathy in the adult population in China: the Beijing eye study. Br J Ophthalmol. 2006;90(9):1087–1090. doi: 10.1136/bjo.2006.096123 16774957 PMC1857376

[35] Wang Q, Wang YX, Wu SL, Chen SH, Yan YN, Yang MC, et al. Ocular axial length and diabetic retinopathy: The Kailuan Eye Study. Invest Ophthalmol Vis Sci. 2019;60(10):3689–3695. doi: 10.1167/iovs.19-27531 31469896

